# Supplementary material for: Investigating the sequence landscape in the Drosophila initiator core promoter element using an enhanced MARZ algorithm
Source: PeerJ. 2023 Jun 22;11:e15597. doi: 10.7717/peerj.15597 (PMC10290830; doi:10.7717/peerj.15597)
Supplement: Supplemental Information 4 — Models were identified as Good (green) or Poor (red) performers based on RZ score. Cut-off values to determine the qualification of an RZ score was determined at each threshold (0, 0.25, 0.50, 0.75 and 1). These cut-off RZ values were chosen to ideally specify three Good and three Poor models at each threshold, although the distribution of scores sometimes resulted in more or fewer models being counted. The Model Number and corresponding gapped models are shown (m = considered nucleotide position, k = ignored nucleotide position). The frequency of the considered nucleotide positions across the sliding window employed by each model is depicted in the final column. [file peerj-11-15597-s004.docx]

| **CPE** | **Threshold** | **Performance** | **Type ID** | **Gapped *n*-mer model** | **Nucleotides considered**  **(shown in red)** |
| --- | --- | --- | --- | --- | --- |
| INR | 0.00 | Poor | 6 | mmkm | 1234­­__  _2345_  __3456  122211 |
|  |  | Poor | 10 | mkmkm | 12345­_  _23456  111111 |
|  |  | Poor | 20 | mkmkkm | 123456  101001 |
|  |  | Good | 15 | mmmmm | 12345_  _23456  122221 |
|  |  | Good | 25 | mmkkmm | 123456  110011 |
|  |  | Good | 31 | mmmmmm | 123456  111111 |
| INR | 0.25 | Poor | 18 | mkkmkm | 123456  100101 |
|  |  | Poor | 22 | mkmmkm | 123456  101101 |
|  |  | Good | 17 | mkkkmm | 123456  100011 |
|  |  | Good | 24 | mmkkkm | 123456  110001 |
|  |  | Good | 25 | mmkkmm | 123456  110011 |
| INR | 0.50 | Poor | 18 | mkkmkm | 123456  100101 |
|  |  | Poor | 22 | mkmmkm | 123456  101101 |
|  |  | Good |  | mmkkm | 12345_  _23456  121011 |
|  |  | Good |  | mmmkm | 12345_  _23456  122111 |
|  |  | Good |  | mmkkmm | 123456  110011 |
|  |  | Good |  | mkmkkm | 123456  101001 |
| INR | 0.75 | Poor | 22 | mkmmkm | 123456  101101 |
|  |  | Poor | 30 | mmmmkm | 123456  111101 |
|  |  | Good | 13 | mmkmm | 12345_  _23456  121121 |
|  |  | Good | 0 | m | 1­­­_____  _2____  __3___  ___4__  ____5_  _____6  111111 |
|  |  | Good | 1 | mm | 12­­­­____  ­_23___  __34__  ___45_  ____56  122221 |
|  |  | Good | 3 | mmm | 123­­___  _234__  __345_  ___456  123321 |
|  |  | Good | 4 | mkkm | 1234­­__  _2345_  __3456  111111 |
|  |  | Good | 6 | mmkm | 1234­­__  _2345_  __3456  122211 |
|  |  | Good | 15 | mmmmm | 12345_  _23456  122221 |
| INR | 1.00 | Poor | 17 | mkkkmm | 123456  100011 |
|  |  | Poor | 20 | mkmkkm | 123456  101001 |
|  |  | Poor | 16 | mkkkkm | 123456  100001 |
|  |  | Good | 26 | mmkmkm | 123456  110101 |
|  |  | Good | 28 | mmmkkm | 123456  111001 |
