# Supplementary figures and images for: Investigating the sequence landscape in the Drosophila initiator core promoter element using an enhanced MARZ algorithm
Source: PeerJ. 2023 Jun 22;11:e15597. doi: 10.7717/peerj.15597 (PMC10290830; doi:10.7717/peerj.15597)

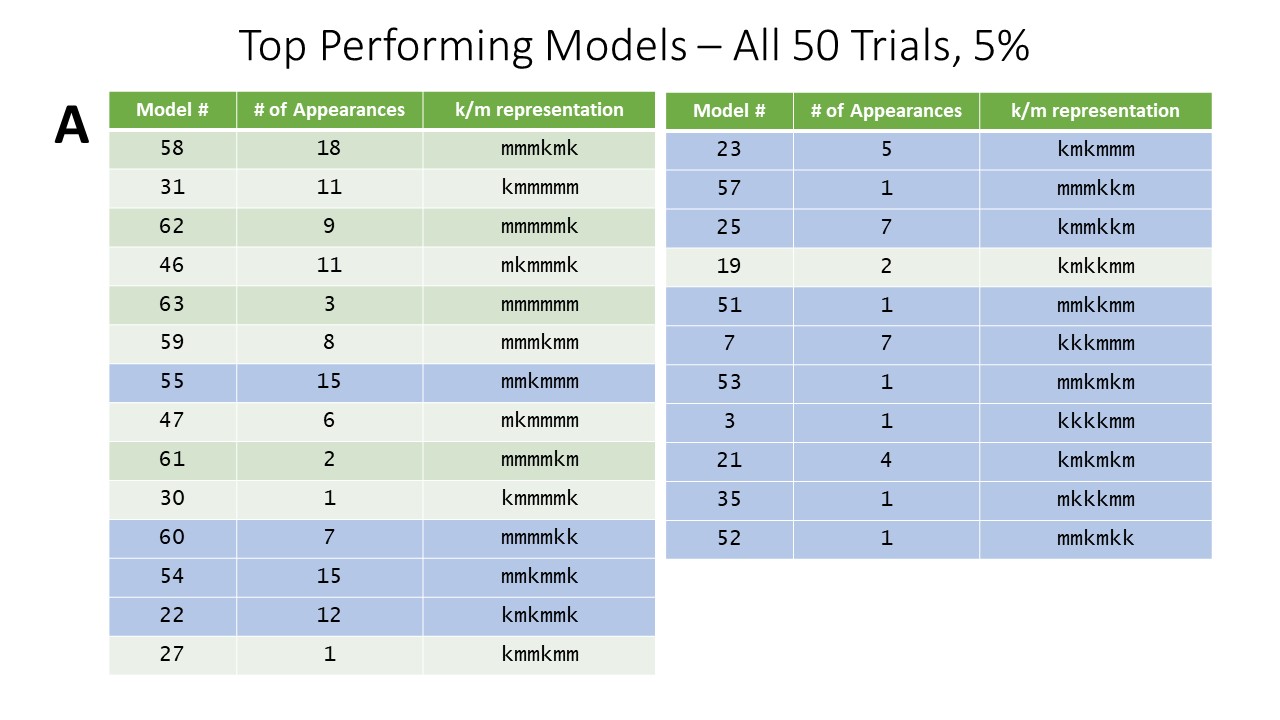


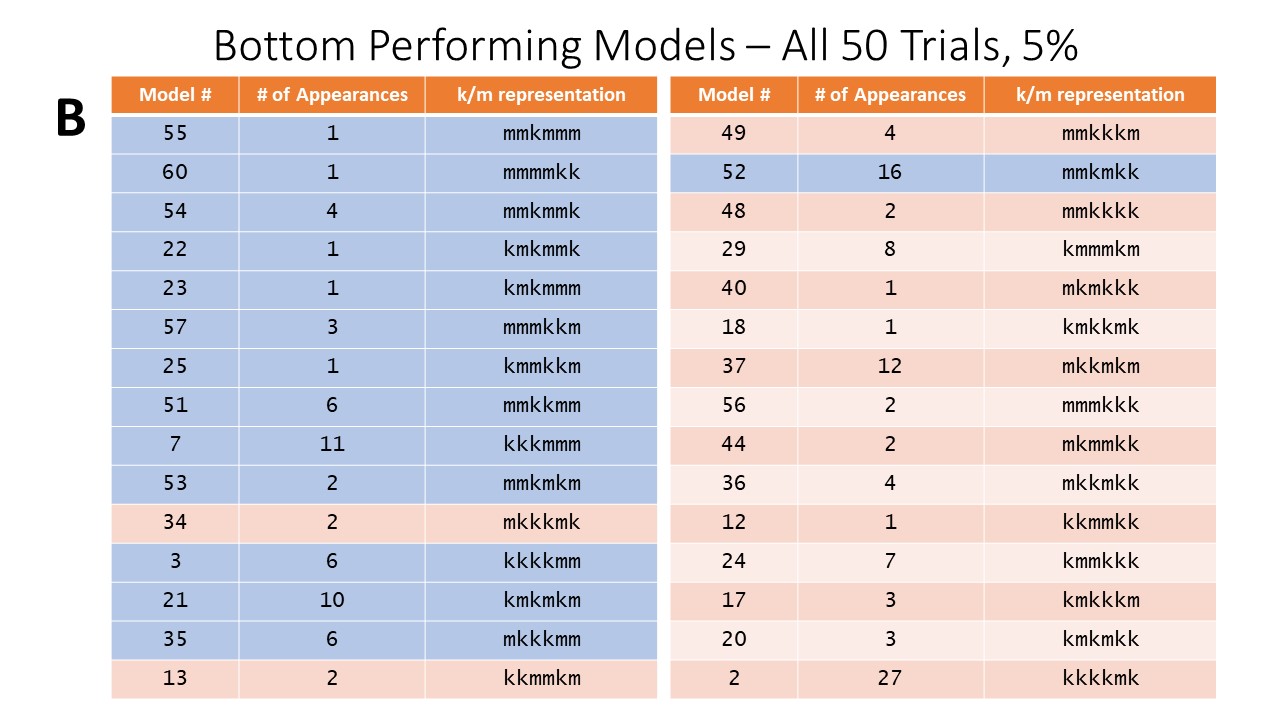

Supplement: Supplemental Information 6 — (A) Top 5% models. The first column is the Model Number, which range from 0 to 63. The second column is the number of times that model appeared in the top 5% of the trials; this number can range from 1 (if it appeared in the top 5% of one of the 50 trials) to 50 (if it appeared in the top 5% of every trial). The third column is the k/m representation of each model. Models that appear in the top 5% of trials are shown in green. If a model also appears at least once in the bottom 5% of one of the trials it is shown in blue. (B) Bottom 5% models. Models that are unique to the bottom 5% are shown in orange. Models that also appear in the top 5% of one of the trials are shown in blue. Both tables are in descending order of average mean true hit ratio. [file peerj-11-15597-s006.docx]

Type ID: 6  
Gapped n-mer model: **mmkm**

Type ID: 10  
Gapped n-mer model: **mkmk**

Type ID: 20  
Gapped n-mer model: **mkmk**

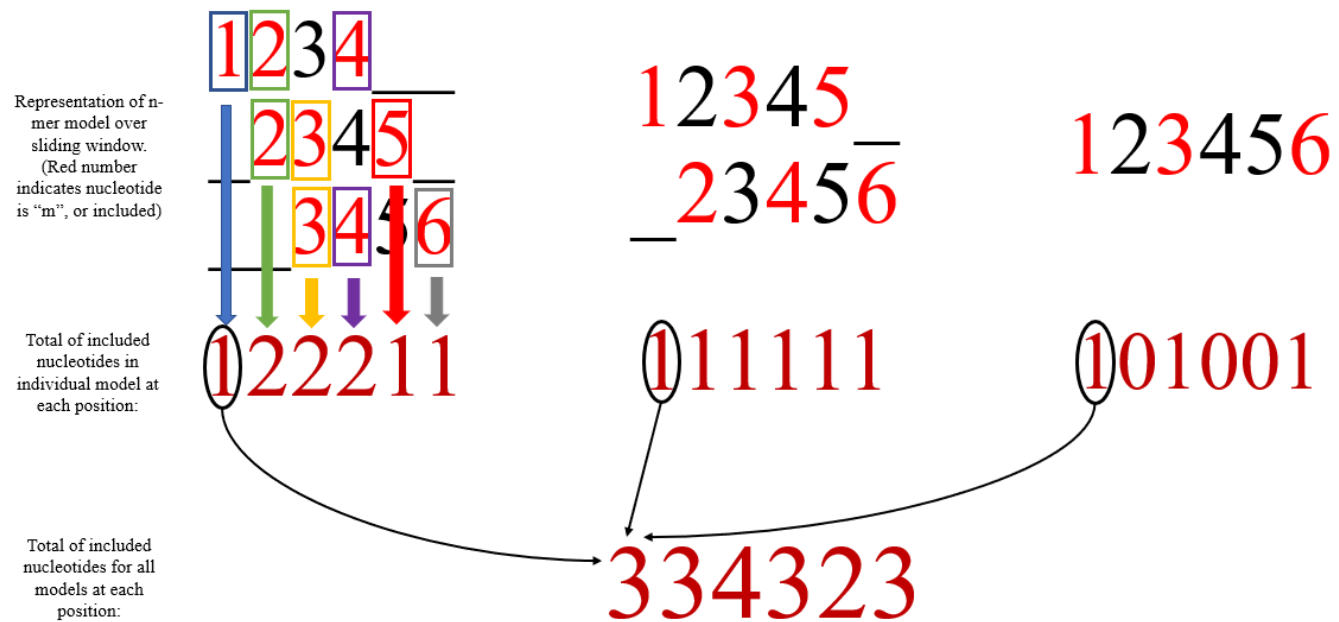

Supplement: Supplemental Information 7 — The method of totaling considered nucleotides, using data for INR threshold 0 poor performance (models 6, 10 and 20) as an example. If a considered nucleotide (red) appears in the sliding window, then a 1 is added to the total for that nucleotide position. The total number of times a position is considered is then summed for all models. [file peerj-11-15597-s007.pdf]
